# Huntington's Disease and Criminal Behaviour: An Exploration of Psychiatric Risk and Management in a High Secure Forensic Unit

**DOI:** 10.1192/bjo.2024.665

**Published:** 2024-08-01

**Authors:** Thomas Gupta-Jessop, Ellice Caldwell-Dunn

**Affiliations:** 1 Forensicare, Melbourne, Australia; 2 Central and North West London NHS Trust, London, United Kingdom

## Abstract

**Aims:**

Huntington’s disease (HD) is an autosomal dominant neurodegenerative disorder characterised by a pathologically prolonged CAG nucleotide sequence in the huntingtin gene (HTT). Neuropsychiatric symptoms such as aggression, depression, impulsivity and psychosis are non-motor signs of HD. The association between HD and criminal behaviour is debated, and evidence lacking. This is particularly relevant in forensic psychiatry, which focusses on the risk assessment of mentally disordered offenders. This manuscript examines the antecedents of offending behaviour in a male diagnosed with HD during admission to a high secure unit, and the evolution of his risk profile from childhood to post-diagnosis. Additionally, through exploration of psychopharmacological management of psychiatric symptoms in HD, this study aims to further our understanding as to how we can best support people with HD in a forensic mental health setting.

**Methods:**

Following review of relevant literature on criminal behaviour in the context of HD, we report the case of a 41-year-old man with a background of dissocial personality traits admitted to a high security unit with symptoms of a delusional disorder; manifesting as paranoia, delusional beliefs and aggression. These were believed to be organically induced within the context of HD, a diagnosis confirmed through genetic testing six months following admission. The patient's symptoms were only partially responsive to first-line antipsychotics; however, good symptomatic control was achieved with clozapine and sodium valproate, enabling step-down to medium secure specialist services.

**Results:**

In HD patients, there may be a challenge of discerning whether offending behaviour relates to prodromal presentation or whether there are pre-existing antisocial attitudes or behaviour; an uncertainty which was present in this case and within the literature. The age of HD onset is inversely correlated with CAG repeat length, and a longer repeat length has been associated with criminal behaviour. This has the potential for use as a marker to determine the time point in which presenting features are attributable to HD. In this case study it was possible to determine through analysis of the CAG repeat length that the delusional disorder was likely linked to the onset of HD; however, dissocial personality traits were not.

**Conclusion:**

A patient's background relating to the life-course persistence of violence, suicidality and psychiatric symptoms in patients with HD informs the process of formulating their risk profile. Changes to the risk profile also reflect the progressing stages of HD. This highlights the need for awareness of how HD may contribute or predispose to criminal behaviour and how interventions could be targeted during critical periods where they benefit most.

